# Effect of NDP-α-MSH on PPAR-γ and –β Expression and Anti-Inflammatory Cytokine Release in Rat Astrocytes and Microglia

**DOI:** 10.1371/journal.pone.0057313

**Published:** 2013-02-26

**Authors:** Lila Carniglia, Daniela Durand, Carla Caruso, Mercedes Lasaga

**Affiliations:** Instituto de Investigaciones Biomédicas, School of Medicine, University of Buenos Aires – CONICET, Buenos Aires, Argentina; Centro Cardiologico Monzino IRCCS, Italy

## Abstract

Brain inflammation plays a central role in numerous brain pathologies. Microglia and astrocytes are the main effector cells that become activated when an inflammatory process takes place within the central nervous system. α-melanocyte-stimulating hormone (α-MSH) is a neuropeptide with proven anti-inflammatory properties. It binds with highest affinity to the melanocortin receptor 4 (MC4R), which is present in astrocytes and upon activation triggers anti-inflammatory pathways. The aim of this research was to identify anti-inflammatory mediators that may participate in the immunomodulatory effects of melanocortins in glial cells. Since peroxisome proliferator-activated receptors (PPARs) have recently been implicated in the modulation of inflammation, we investigated the effect of an α-MSH analog, [Nle^4^, D-Phe^7^]-α-MSH (NDP-α-MSH), on PPAR-β and PPAR-γ gene and protein expression in rat primary astrocytes and microglia. We initially demonstrated that rat primary microglia express MC4R and showed that treatment with NDP-α-MSH increases PPAR-γ protein levels and strongly decreases PPAR-β levels in both astrocytes and microglia. We also showed that extracellular signal-regulated kinase 1/2 (ERK1/2)–mediated signaling is partially involved in these effects in a cell-specific fashion. Finally, we showed that NDP-α-MSH stimulates the release of the anti-inflammatory cytokines IL-10 and TGF-β from microglia and astrocytes, respectively. The presented data suggest a role for IL-10 and TGF-β in the protective action of melanocortins and a connection between MC4R pathway and that of the nuclear receptor PPAR-γ. This is the first report providing evidence that MC4R is expressed in rat primary microglia and that melanocortins modulate PPAR levels in glial cells. Our findings provide new insights into the mechanisms underlying the activation of glial MC4R and open perspectives for new therapeutic strategies for the treatment of inflammation-mediated brain diseases.

## Introduction

Astrocytes are the most abundant cell type in the central nervous system (CNS). Along with microglia, the resident macrophages of the CNS, they play a major role in the regulation of the inflammatory responses, since they can become activated by specific pathogenic stimuli or damage signals and modify the expression of numerous inflammatory mediators [1].

The α-melanocyte-stimulating hormone (α-MSH) is a member of the melanocortin family, a group of peptides derived from the post-translational cleavage of their precursor pro-opiomelanocortin (POMC), which occurs in a tissue-specific manner. α-MSH is produced mainly in neurons of the arcuate nucleus of the hypothalamus, in the intermediate lobe of the anterior pituitary and in discrete groups of neurons placed between the hypothalamus and the medulla [2]. POMC mRNA has also been detected in lymphocytes, monocytes, keratinocytes and melanocytes [3], and α-MSH was shown to be released by a microglial cell line [4]. Melanocortins exert their action through the activation of five melanocortin receptor subtypes (MC1R to MC5R), which are coupled to G-protein and upon activation induce the increase of cyclic AMP (cAMP) levels [5]. Several studies have also reported the activation of the extracellular signal-regulated kinase 1/2 (ERK1/2) pathway upon MCR stimulation [6], [7], [8]. Particularly, Vongs et al. have shown that the α-MSH analog [Nle^4^, D-Phe^7^]-α-MSH (NDP-α-MSH) induces ERK-1/2 activation through MC4R [9]. In the CNS the most widely distributed subtype of melanocortin receptor is MC4R. We and others have demonstrated that MC4R is the only MCR expressed in rat primary astrocytes [10], [11]. Lindberg et al. detected mRNA expression of MC1R, MC3R, MC4R and MC5R in a human microglial cell line [12] and, in a recent study published by Malik et al., the authors show MC4R immunoreactivity in rat brain tissue sections in glial cells, including microglia [13]. However, the expression pattern of MCRs in rat primary microglia has not yet been studied.

Protective effects of α-MSH, acting through MC4R, have been reported in several *in vivo* models of ischemia and inflammation [14], as well as in a variety of *in vitro* systems where it has been shown to down-regulate the production and release of inflammatory mediators [5]. Particularly, we have previously shown that α-MSH reduces the production of nitric oxide (NO) and prostaglandins (PGs) induced by lipopolysaccharide (LPS) plus interferon-γ (IFN-γ) in astrocytes [10]. We have also shown that α-MSH, acting via MC4R, inhibits the expression of inducible nitric oxide synthase (iNOS) and cyclooxigenase (COX-2) in rat hypothalamus [15]. Furthermore, we have demonstrated that α-MSH and its synthetic analog NDP-α-MSH, through MC4R activation, induce production of cAMP and stimulate the cAMP- protein kinase A-cAMP responsive element binding protein (cAMP-PKA-CREB) pathway in astrocytes [16]. NDP-α-MSH, acting through MC4R, modulates inflammatory and apoptotic cascades in a model of global cerebral ischemia in gerbils [17] and activation of MC4R improves survival in a model of severe haemorrhagic shock in rats [18]. Furthermore, NDP-α-MSH protects against traumatic brain injury through MC4R activation [19]. These data support the notion that protective effects of melanocortins in the CNS are mediated mainly by MC4R activation. In a microglial cell line, Delgado et al. showed that α-MSH inhibits TNF-α, interleukin-6 (IL-6) and NO production induced by LPS plus IFN-γ, and that α-MSH is released from microglia acting as an autocrine regulatory factor [4]. However, the MCR subtype involved in the anti-inflammatory action of α-MSH on microglial cells is yet to be determined.

Peroxisome proliferator-activated receptors (PPARs) are members of the nuclear receptor superfamily of ligand-activated transcription factors. Upon ligand binding, these receptors form heterodimers with retinoid X receptor and then bind specific DNA sequences thereby modulating the expression of their target genes. To date, three isotypes have been identified: α, β (a.k.a. δ) and γ, each with a specific tissue distribution and ligand specificity, all three found in the brain [20]. Recent studies have implicated PPARs in the control of inflammation; their protective effects have been described in several *in vivo* models of CNS disorders with an inflammatory component [21], [22], [23]. In glial cells, PPARs modulate the production of pro-inflammatory mediators such as NO, TNF-α, IL-1β, IL-6, iNOS and COX-2 [22], [24].

In this study, we sought to identify possible mechanisms involved in the anti-inflammatory actions of melanocortins in glial cells. We first studied the expression of the different MCRs in rat microglial cells. We then investigated the effect of NDP-α-MSH on PPAR-γ and PPAR-β gene and protein expression and the possible participation of the ERK-1/2 signaling pathway in these effects. Finally, we examined the effect of NDP-α-MSH on the release of the anti-inflammatory cytokines IL-10 and TGF-β in both cell types and the possible involvement of PPARs in these effects.

## Materials and Methods

### Ethics Statement

Experimental procedures were approved by the Institutional Committee for the Care and Use of Laboratory Animals (CICUAL) of the School of Medicine, University of Buenos Aires, Argentina (resolution n° 096/2010) and were carried out in compliance with the guidelines of the NIH Guide for the Care and Use of Laboratory Animals.

### Reagents

NDP-α-MSH was obtained from Bachem (CA, USA). HS024 and GW9662 were purchased from Tocris Bioscience (MO, USA). PD98059 was purchased from Merck KGaA (Darmstadt, Germany). Fetal bovine serum (FBS) was obtained from PAA laboratories GmBH (Pasching, Austria). DMEM/F-12, DMEM, MEM, antibiotics and all RT-PCR reagents were purchased from Invitrogen Life technologies (CA, USA). Biotinylated anti-rabbit antibody was from Millipore (MA, USA). PPAR-γ (H-100), PPAR-β (H-74) and Glyceraldehyde 3- phosphate dehydrogenase (GAPDH, FL-335) antibodies were from Santa Cruz Biotechnologies (CA, USA). MC4R antibody was purchased from Cayman Chemical (MI, USA). MC4R primers were from Integrated DNA Technologies, Inc. (Coralville, IA). All other media and supplements were obtained from Sigma-Aldrich Corporation, unless specified otherwise.

### Primary Astrocyte and Microglial Cultures

1–2 days old Wistar rat pups were decapitated and their brains were removed and freed from meninges. Cells were mechanically dispersed and seeded in previously poly-L-lysine-coated culture flasks, and maintained in DMEM/F-12 supplemented with 10% FBS, 50 µg/ml streptomycin and 50 U penicillin, at 37°C in 5% CO_2_. Medium was replaced twice a week. After 11–14 days, microglial cells were detached from astrocytes by shaking for 1–2 h at 110 rpm, at 33°C in a Thermo Scientific Orbital Shaker (Thermo Fischer Scientific, Germany). Supernatants were collected and centrifuged, and microglia was seeded in uncoated plates in supplemented DMEM and left to stabilize for 24 h at 37°C in 5% CO_2_ before adding the drugs in fresh supplemented DMEM containing 2% FBS and 2 mM L-glutamine. Cells were stained with a microglial marker (anti-rat CD11b monoclonal antibody, OX-42, Millipore) to assess their purity, which was close to 93%.

Astroglial cultures were separated from oligodendrocytes by shaking once every 2–3 days for 24 h at 180 rpm, at 33°C in a Thermo Scientific Orbital Shaker (Thermo Fischer Scientific, Germany). After 3 weeks of culture astrocytes were trypsinized, subcultured and left to stabilize for 2–3 days at 37°C in 5% CO_2_. Then they were incubated with the drugs in MEM supplemented with 2% FBS, 6 mM L-glutamine, 50 µg/ml streptomycin and 50 U penicillin. Cultures were routinely close to 95% astrocytes, as assessed by immunostaining with GFAP monoclonal antibody (Millipore, MA, USA), as previously described [10].

### Immunocytochemistry for MC4R

Cells were washed in phosphate buffered saline (PBS) and fixed in PBS–formaldehyde 4% for 15 min at room temperature (RT). Then cells were incubated in blocking solution containing 10% donkey serum, 5% rat serum and 0.2 mL/mL avidin solution in PBS for 1 h at RT. Subsequently, cells were incubated overnight at 4°C with the primary antibody against MC4R (1∶100) in blocking solution containing 1% donkey serum, 0.5% rat serum and 0.2 mL/mL biotin solution. After rinsing, slides were incubated with a biotin-conjugated donkey anti-rabbit secondary antibody (1∶200) in blocking solution containing 1% donkey serum for 1 h at RT. Cells were washed and incubated with FITC-conjugated avidin (1∶400 in 10 mM HEPES, pH 7.9) for 30 min at RT, washed in PBS and mounted in mounting medium Vectashield (Vector Laboratories). Staining was visualized in a fluorescence microscope (Axiophot; Carl Zeiss, Jena, Germany). Negative control slides were incubated with blocking solution instead of primary antibody.

### Reverse-transcription Polymerase Chain Reaction (RT-PCR)

Total DNA and RNA was extracted using Trizol reagent (Invitrogen Life technologies, CA, USA), following the manufacturer’s instructions. 5 µg of RNA for astrocyte samples or 3 µg of RNA for microglial samples were treated with 1 U DNAse (Promega Corp., Madison, WI) and reverse-transcribed as described before [15]. Synthetic oligonucleotides used for amplification of MCRs were: MC1R forward: 5′-TCTGCTGCCTGGCCCTGTCTGA-3′, MC1R reverse: 5′-TGCTGGCACGCTCTCGTGAACA-3′, MC3R forward: 5′-AGCAACCGGAGTGGCAGT-3′, MC3R reverse: 5′-GGCCACGATCAAGGAGAG-3′, MC4R forward: 5′-GGCTTCACATTAAGAGGATCGCT-3′, MC4R reverse: 5′-TTTATGGAACTCCATAGCGCCC-3′, MC5R forward: 5′-TCTTCCAGCGATGAACTCCT-3′, MC5R reverse: 5′-AAGCAAGCGCAATAGACGTT-3′. PCR products were electrophoresed in a 2% agarose gel and visualized by ethidium bromide staining.

### Real-time Quantitative PCR (qPCR)

Products of the Reverse -Transcription reaction were amplified using specific primers and SYBR Green Master Mix (Invitrogen Life Technologies) on a StepOneTM Real-Time PCR System (Applied Biosystems). For astrocyte samples primers were used at a concentration of 300 ηM for PPAR-γ and HPRT, and 900 ηM for PPAR-β. For microglial samples primers were used at a concentration of 300 ηM for PPAR-γ and HPRT, and 450 ηM for PPAR-β. Synthetic oligonucleotides used for qPCR were: PPAR-γ forward: 5′-CCCACCAACTTCGGAATCAG -3′, PPAR-γ reverse: 5′-GGAATGGGAGTGGTCATCCA-3′, PPAR-β forward: 5′- CTCCTGCTGACTGACAGATG-3′, PPAR-β reverse: 5′- TCTCCTCCTGTGGCTGTTC-3′, HPRT forward: 5′-CTCATGGACTGATTATGGACAGGAC-3′, HPRT reverse: 5′-CAGGTCAGCAAAGAACTTATAGCC-3′. PCR conditions were: denaturation at 95°C for 1 min, followed by 40 cycles of 95°C for 1 min and 60°C for 1 min. The PCR product specificity was verified by a melting curve analysis. No-RT controls were performed by omitting addition of the reverse transcriptase enzyme, and no-template controls were performed by addition of nuclease free water instead of cDNA. Gene expression was normalized to the endogenous reference gene HPRT by the relative quantitative method (ΔΔCt) [33] using Step-One Software (Applied Biosystems), and expressed as fold-changes relative to the control group.

### Protein Extraction and Western Blotting

Cells were scrapped in cold PBS and lysed in lysis buffer (50 mM Tris-HCl pH 7.4, 1 mM EDTA, 150 mM NaCl, 1% NP-40, 1 mM PMSF, 1 µg/mL Aprotinin, 1 µg/mL Leupeptin, 1 µg/mL Pepstatin A, 10 mM NaF, 1 mM Na_3_VO_4_). Protein levels were quantified with Bradford’s reagent and protein samples (30–40 µg) were subjected to electrophoretic separation on a 10% SDS-polyacrilamide gel and then electrotransferred to a polyvinylidene difluoride membrane. Blots were blocked for 1.5 h at RT in 5% non-fat dry milk-Tris-buffered saline- 0.1% Tween-20 (TBST) and incubated overnight with the appropriate primary antibody dilution (PPAR-γ 1∶500, PPAR-β 1∶500, GAPDH 1∶10000) in 5% non-fat dry milk-TBST at 4°C. This was followed by incubation for 1 h at RT with the respective biotinylated secondary antibody, and 1 h incubation at RT with streptavidin-peroxidase (Millipore, MA, USA). Immunoreactivity was detected by chemiluminescence (Bio-Lumina, PB-L, Prod. Bio Lógicos, Argentina) and semi-quantified using SCION Image software. Data were normalized to the internal control GAPDH and expressed as fold-changes relative to unstimulated cells.

### Cytokine Assays

Cytokine release was assessed by ELISA, using commercial kits (IL-10: BD Biosciences; TGF-β: Invitrogen). Assays were performed following the manufacturer’s instructions, with the exception of TGF-β assay buffer which was PBS with 2% heat-inactivated FBS and 0.1% Tween-20.

### Statistical Analysis

Results are expressed as mean ± SEM of at least three independent experiments. Data were analyzed by one sample t test, unpaired Student’s t test or one-way analysis of variance (ANOVA) followed by Dunnet or Bonferroni Multiple Comparisons Test, as required by the experimental design. GraphPad Prism 5 Software was used (GraphPad Software, CA, USA). Differences with a value of *p*<0.05 were considered statistically significant.

## Results

In the present work we focused on the study of possible mediators of melanocortins’ anti-inflammatory actions on glial cells. Given its greater enzymatic stability we used the synthetic compound NDP-α-MSH [34], an analog of α-MSH that can bind all MCRs but has greater affinity for MC4R and MC5R [35].

### Melanocortin 4 Receptor Expression in Rat Microglia

**Figure 1:**
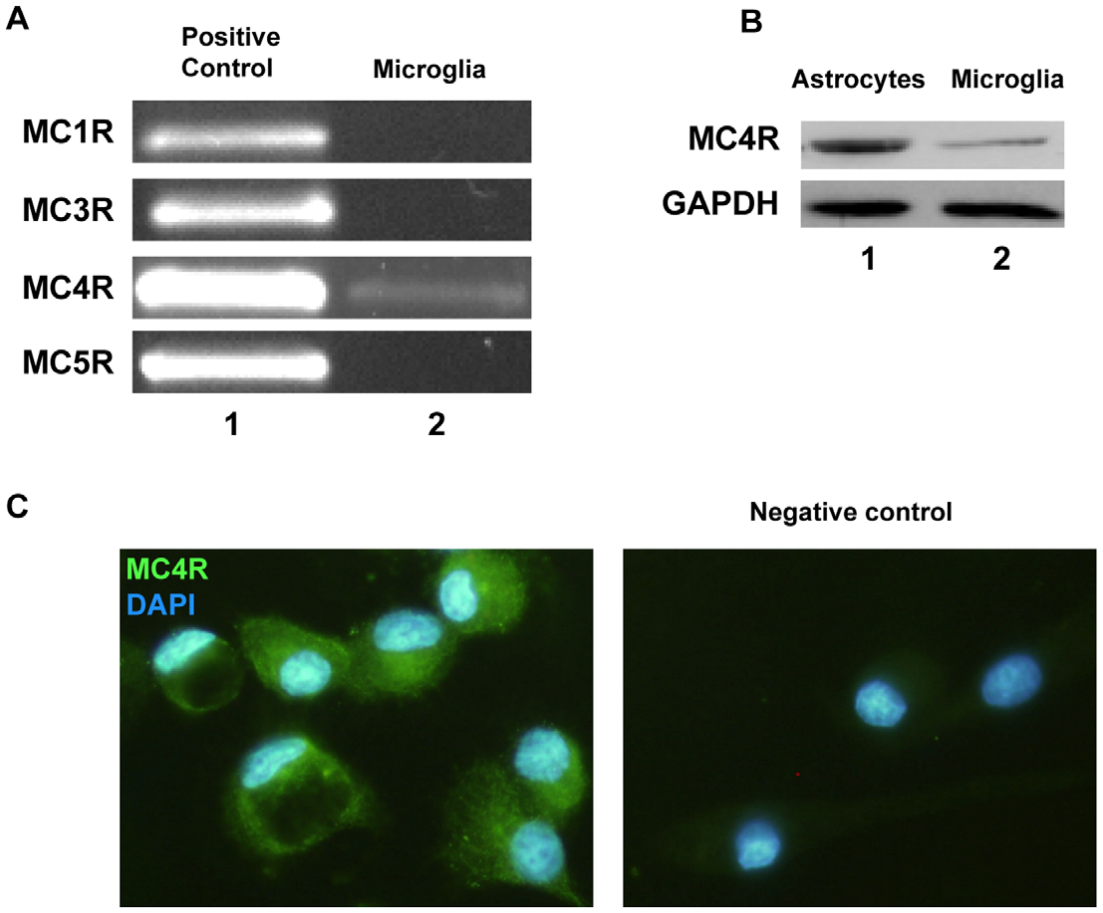
Melanocortin 4 receptor expression in rat microglia. (A) MCR mRNA expression was assessed by RT-PCR using total mRNA from untreated microglial cells. Genomic DNA was used as a positive control (lane 1). A band close to 595 pb corresponding to MC4R amplification product was detected in primary cultured microglial cells (lane 2, MC4R). We did not detect expression of the other MCR subtypes in these cells (lane 2). (B) MC4R protein expression was evaluated by western blot. Total protein sample from astrocytes was used as a positive control. Results show the presence of a band of nearly 50 KDa in both astrocytes (lane 1) and microglia (lane 2). (C) MC4R was detected in primary microglia by immunocytochemistry. The left panel shows MC4R immunoreactivity in microglial cells. Nuclei were stained with DAPI to identify individual cells. The negative control is shown in the right panel.

Our first aim was to study MCR mRNA expression in rat microglial cells by RT-PCR using total mRNA from untreated primary microglial cultures. RT-PCR for MC4R yielded an amplification product near 595 bp (as expected for MC4R) in microglial cells. However, we did not find expression of MC1R, MC3R or MC5R in these cells (Fig. 1.A). Protein expression was evaluated by western blot using a specific primary antibody against MC4R (Fig. 1.B). The blot shows the presence of a band of nearly 50 KDa in the microglial sample, as well as in astrocytes (positive control). We also detected microglial MC4R immunoreactivity by immunocytochemistry (Fig. 1.C). Our data confirm that rat primary cultured microglial cells express MC4R, while mRNA expression of the other MCR subtypes was not detected in these cells.

### NDP-α-MSH Modulates PPAR-γ and PPAR-β Expression in Microglia and Astrocytes

**Figure 2:**
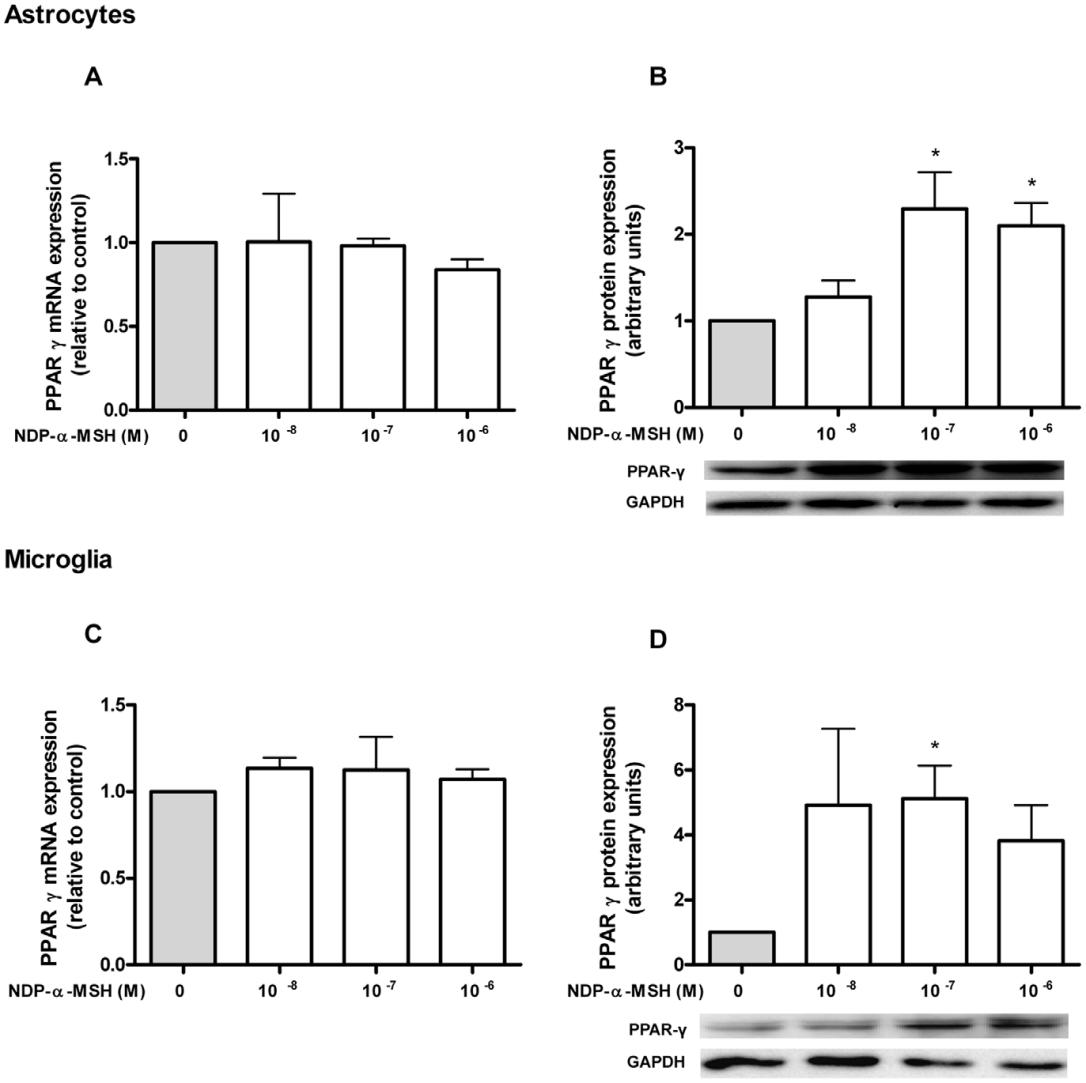
PPAR-γ expression in rat astrocytes and microglia. (A and C) Gene expression was studied by RT-qPCR as described in Materials and Methods. No significant change in PPAR-γ mRNA levels was observed after 24 h of incubation with NDP-α-MSH in both cell types. (B and D) Protein expression of PPAR-γ was assessed by western blot after 24 h of treatment. NDP-α-MSH induced the expression of PPAR-γ protein in both cell types. Data were analyzed by one sample t Test and are expressed as mean ± SEM. *p<0.05 vs. control group.

**Figure 3:**
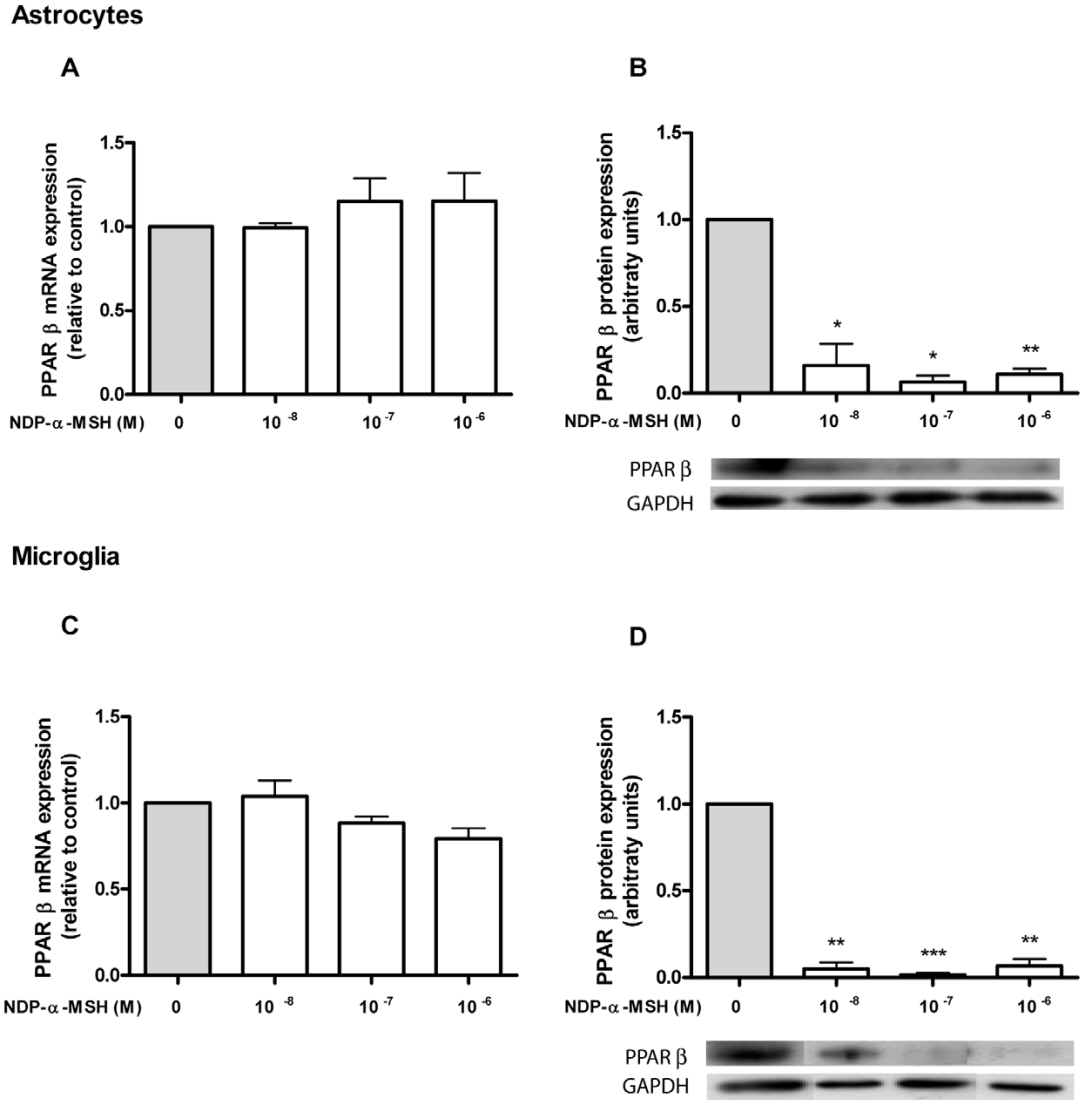
PPAR-β expression in rat astrocytes and microglia. (A and C) Gene expression was studied by real time quantitative RT-PCR as described in Materials and Methods. No significant change in PPAR-β mRNA levels was detected after 24 h of incubation with NDP-α-MSH in both cell types. (B and D) Protein expression of PPAR-β was assessed by western blot after 24 h of treatment. NDP-α-MSH markedly decreased PPAR-β protein levels in both cell types. Data were analyzed by one sample t Test and are expressed as mean ± SEM. ***p<0.001, **p<0.01, *p<0.05 vs. control group.

PPARs are known to be expressed within the brain [20]. Our aim was to assess the expression of PPAR-γ and -β in rat astrocytes and microglia and to study their modulation by NDP-α-MSH. We detected basal mRNA expression of PPAR-γ and PPAR-β, but we did not find a significant change in mRNA levels after 24 h of treatment with NDP-α-MSH, both in astrocytes (Fig. 2.A and 3.A) and in microglia (Fig. 2.C and 3.C). We then decided to investigate the effect of NDP-α-MSH on the receptor’s protein expression, since PPARs can be regulated at the post-transcriptional level without changes being observed in their gene expression [36]. PPAR-γ and PPAR-β protein expression was assessed by western blot after 24 h of treatment. NDP-α-MSH significantly induced the expression of PPAR-γ in astrocytes (Fig. 2.B) and in microglia (Fig. 2.D) whereas it caused a remarkable decrease of PPAR-β protein levels in both cell types (Fig. 3.B and 3.D).

### ERK-1/2 Participates in the NDP-α-MSH-induced Modulation of PPAR Protein Expression in Astrocytes and Microglia

**Figure 4:**
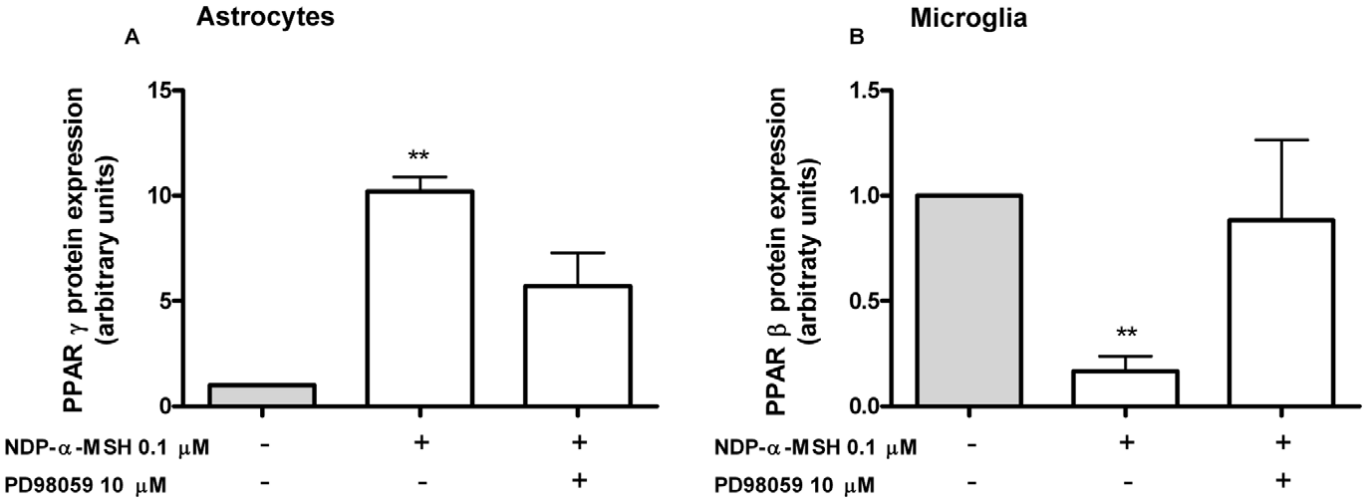
ERK-1/2 partially mediates the effect of NDP-α-MSH on PPAR expression. Cells were treated for 15 min with 10µM PD98059 and then 0.1 µM NDP-α-MSH was added to the culture medium. Cells were harvested after 24 h of incubation and PPAR protein levels were assessed by western blot. (A) The effect of NDP-α-MSH over PPAR-γ expression was not observed in the presence of PD98059 in astrocytes. (B) Similarly, the effect of NDP-α-MSH on PPAR-β levels was not observed in the presence of PD98059 in microglial cells. Data were analyzed by one sample t Test and are expressed as mean ± SEM. **p<0.01 vs. control group.

The finding that NDP-α-MSH modulates PPAR protein expression led to the question of what are the mechanisms involved in this modulation. Since stimulation of all MCRs leads to activation of ERK-1/2 [6], [7], [8], [9] and this kinase has been previously linked to the regulation of PPAR activity [37], we evaluated whether ERK-1/2 activation is involved in NDP-α-MSH-induced modulation of PPAR expression in our system. For this, the effect of the MCR agonist was evaluated in the presence of the MEK inhibitor PD98059, which inhibits the ERK-1/2 pathway [38]. The stimulatory effect of NDP-α-MSH on PPAR-γ expression was not observed in the presence of PD98059 in astrocytes (Fig. 4.A). Similarly, the decrease in PPAR-β levels caused by NDP-α-MSH treatment was not observed in the presence of PD98059 in microglial cells (Fig. 4.B). The inhibitor had no effect neither on NDP-α-MSH modulation of PPAR-γ expression in microglial cells nor on NDP-α-MSH modulation of PPAR-β expression in astrocytes (data not shown). Our data suggest a cell-specific role for ERK-1/2-mediated signaling, in which this kinase would be at least partially mediating the NDP-α-MSH-induction of PPAR-γ expression in astrocytes, and the NDP-α-MSH-induced reduction of PPAR-β levels in microglial cells.

### NDP-α-MSH Induces IL-10 Release from Microglial Cells and TGF-β Release from Astrocytes

**Figure 5:**
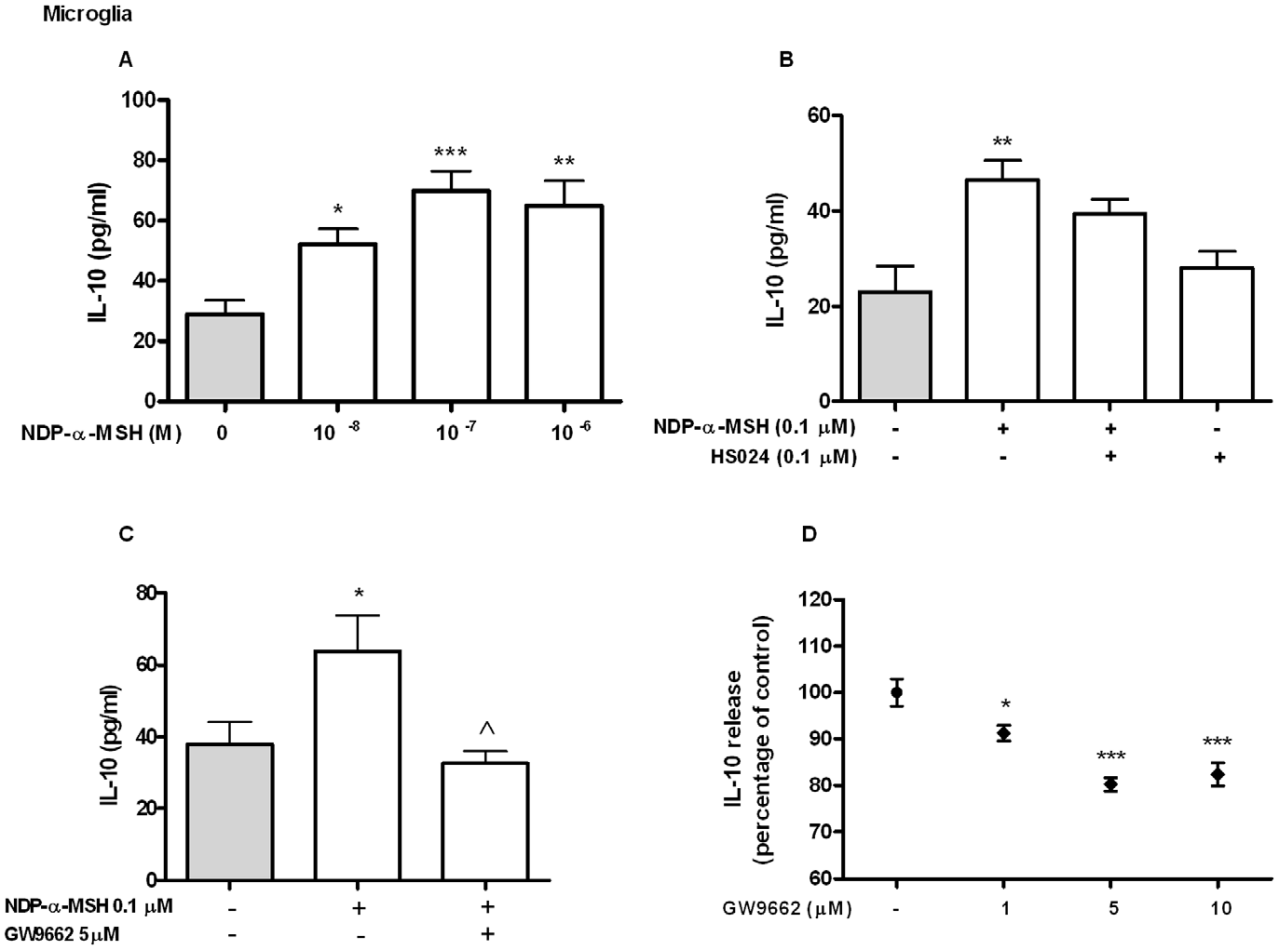
IL-10 release from rat microglia. IL-10 levels in culture supernatants were quantified by ELISA after 24 h of treatment. (A) NDP-α-MSH induced IL-10 release from microglial cells. The stimulatory effect of NDP-α-MSH is not seen in the presence of the MC4R antagonist HS024 (B) or of the PPAR-γ antagonist GW9662 (C). (D) GW9662 has an inhibitory effect *per se* on IL-10 release from microglia. Data were analyzed by one way ANOVA followed by Dunnett’s or Bonferroni’s post test and are expressed as mean ± SEM. ***p<0.001, **p<0.01, *p<0.05 vs. control group, ^∧^ *p*<0.05 vs. NDP-α-MSH.

**Figure 6:**
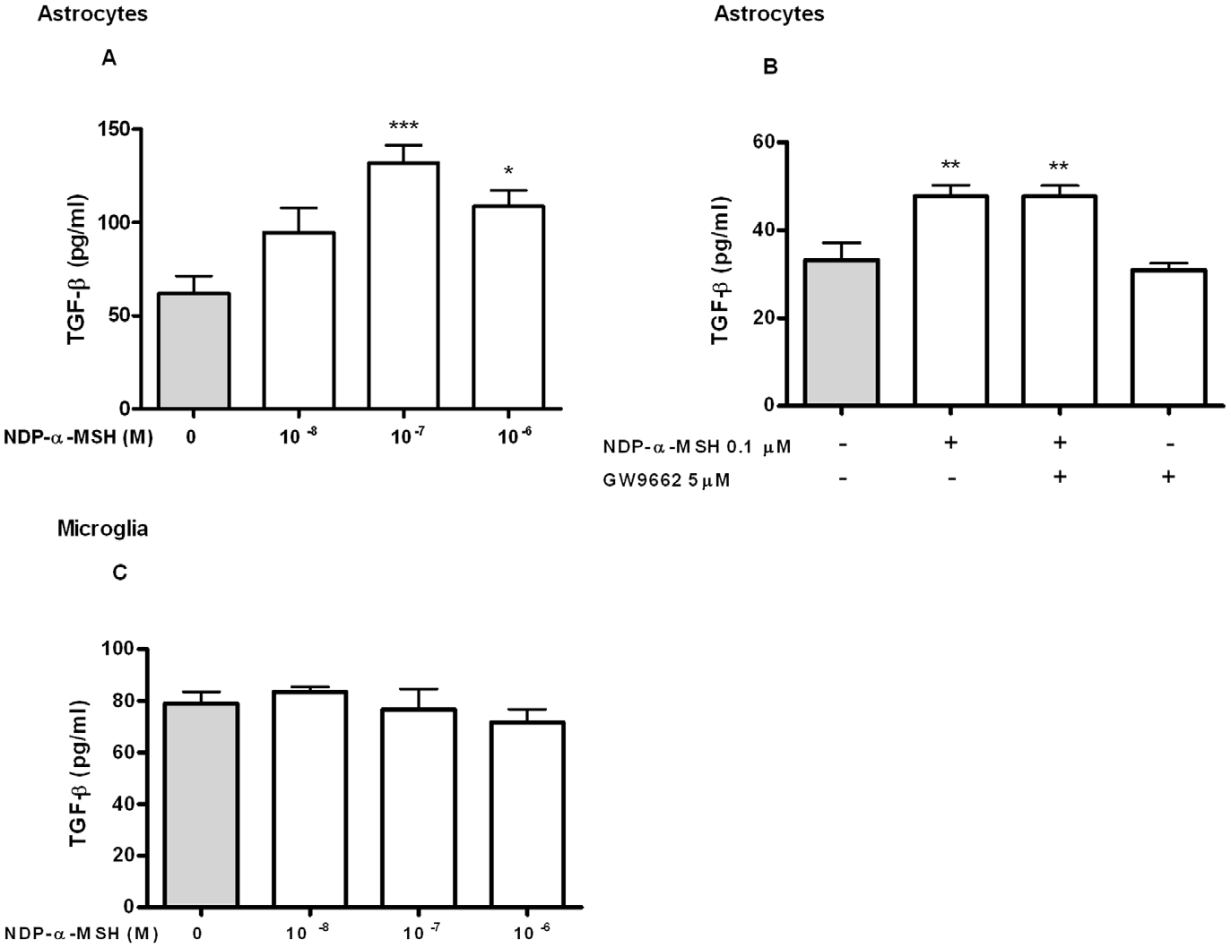
TGF-β release from rat astrocytes. TGF-β levels in culture supernatants were quantified by ELISA after 6 h of treatment. (A) NDP-α-MSH induced TGF-β release from astrocytes. (B) Treatment with 5 µM GW9662 had no effect on NDP-α-MSH-induced TGF-β release. (C) NDP-α-MSH had no effect on TGF-β release from microglia. Data were analyzed by one way ANOVA and are expressed as mean ± SEM. ***p<0.001, **p<0.01, *p<0.05 vs. control group.

Our main interest was to study the modulation of anti-inflammatory mediators by NDP-α-MSH in astrocytes and microglial cells. To determine the effect of NDP-α-MSH on IL-10 release culture supernatants were assayed by ELISA after 24 h of treatment. NDP-α-MSH induced IL-10 release from microglia (Fig. 5.A). The stimulatory effect of NDP-α-MSH was not observed in the presence of the MC4R selective antagonist HS024 [39] (Fig. 5.B). We also found that in the presence of the PPAR-γ antagonist GW9662, NDP-α-MSH did not induce IL-10 release from microglial cells (Fig. 5.C). However, GW9662 decreased IL-10 release *per se* from these cells (Fig. 5.D). On the other hand, IL-10 levels in the supernatant of primary cultured astrocytes were not detectable in the untreated group and we did not observe an effect of NDP-α-MSH on the release of this cytokine in these cells (data not shown). To asses the effect of the MCR agonist on TGF-β release, culture supernatants were tested by ELISA after 6 h of treatment. NDP-α-MSH significantly induced TGF-β release from astrocytes (Fig. 6.A). The PPAR-γ antagonist GW9662 had no effect over NDP-α-MSH-induced TGF-β release in astrocytes (Fig. 6.B). On the other hand, TGF-β release from microglial cells was unaffected by the treatment with NDP-α-MSH (Fig. 6.C).

## Discussion

The data presented herein describe for the first time that activation of MC4R modulates PPAR levels in rat astrocytes and microglia. Our studies demonstrate that NDP-α-MSH treatment increases PPAR-γ levels whereas it decreases PPAR-β levels in both cell types. Moreover, we observed that NDP-α-MSH exerts cell-specific effects, increasing IL-10 release from microglia and TGF-β release from astrocytes. Thus, we describe novel mechanisms following MC4R activation that may be involved in the anti-inflammatory effects of melanocortins in glial cells.Inflammation has been implicated in the pathogenesis of several neurological disorders such as Alzheimeŕs disease, Parkinsońs disease, Huntingtońs disease and Experimental Autoimmune Encephalomyelitis (EAE) [40]. Melanocortin’s protective effects in models of disease with an inflammatory component have been broadly described, both in the periphery and in the CNS [14]. The main candidates for mediating melanocortin’s anti-inflammatory action in the brain are MC3 and MC4 receptors since MC3/MC4R antagonists block the effect of α-MSH on the production of pro-inflammatory cytokines in the CNS [10], [41]. Recently, agonists of MC4R have emerged as promising candidates for the treatment of brain disorders [42]. NDP-α-MSH was found to exert a strong neuroprotection, through the activation of MC4R, against damage following cerebral ischemia and traumatic brain injury (for review see [42]). On the other hand, a selective MC3R agonist did not show protective effects in models of middle cerebral artery occlusion [43] or global cerebral ischemia [17], which supports MC4R as the main mediator of NDP-α-MSH protective effects in the brain. In microglial cells previous studies have demonstrated that α-MSH inhibits TNF-α, NO and IL-6 production induced by distinct pro-inflammatory agents [4], [44], [45] in a concentration-related fashion. Lindberg et al. detected mRNA expression of MC1R, MC3R, MC4R and MC5R in a human microglial cell line [12]. However, receptor expression often varies with cell lines and primary cultured cells and, to our knowledge, there are no published studies on MCR expression in primary microglial cells. Here, we report for the first time the expression of MC4R in rat primary microglia. These data plus the fact that this receptor is the only MCR subtype expressed in rat primary astrocytes [10], [11], further support MC4R as a promising target for modulation of glial activation by melanocortins.This study is also the first to report that a MC4R agonist can induce the protein expression of PPAR-γ in glial cells. Although previous studies have shown that activation of central MC3R and MC4R reduces PPAR-γ gene expression in hepatocytes [46] and Agouti, an endogenous MC1R antagonist, increases PPAR-γ levels in adipocytes [47], a very recent study has demonstrated that α-MSH induces both PPAR-γ upregulation and activation in B16-F10 melanoma cells [48]. In the past few years, accumulating evidence has implicated PPARs in the regulation of inflammatory processes in the CNS [49]. Also, activation of PPAR-γ has been found to protect cortical neurons against NO and hydrogen peroxide-dependent toxicity [50] and PPAR-γ agonists were found to inhibit the release of pro-inflammatory cytokines by microglial cells and astrocytes [51]. Thus, it is possible that PPAR-γ could be taking part in the anti-inflammatory effects of NDP-α-MSH in the CNS.

Our present data also indicate that NDP-α-MSH reduces PPAR-β levels in both astrocytes and microglia. To the best of our knowledge, there is no published data on the effect of melanocortins on PPAR-β expression or activity. Compared to the other isotypes much less is known about the role of PPAR-β in the regulation of inflammatory responses. Anti-inflammatory actions of PPAR-β agonists have been demonstrated in microglia and astrocytes [23] and in a model of focal cerebral ischemia [52]. The fact that NDP-α-MSH treatment results in opposite effects on PPAR-β and PPAR-γ protein expression is not, however, unexpected. This kind of inverse relationship between PPARs has been observed previously. Jana and Pahan showed that LPS increases PPAR-γ expression whereas it decreases that of PPAR-β in primary human astrocytes and microglia, and that treatment with a PPAR-α agonist increases PPAR-β and decreases PPAR-γ expression in LPS-activated astrocytes and microglia [53]. Moreover, Aleshin et al. have demonstrated that both PPAR-γ and PPAR-α influence PPAR-β expression and activity in primary astrocytes [24]. The authors point out that it is not only the absolute levels of expression of the different PPAR isotypes but also their ratio that will ultimately control their activity. Thus, it is likely that a reciprocal modulation of expression between PPAR-γ and PPAR-β may be occurring in our system as well.

Unexpectedly, we found that the effect of NDP-α-MSH on PPAR protein expression was not accompanied by a concomitant change in PPAR’s mRNA levels. However, post-transcriptional regulation of PPARs without observing changes in their mRNA levels has been reported before [36], [54]. Indeed, PPARs can undergo a number of post-translational modifications such as phosphorylation, sumoylation, ubiquitination and nitration with cell and context-specific consequences [55]. It is plausible that the changes observed in PPAR’s protein expression in our system might be due to post-transcriptional mechanisms affecting translational efficiency or protein stability and turnover. Further study is needed to clarify this issue.

We found that ERK-1/2 signaling mediates the stimulatory effect of NDP-α-MSH on PPAR-γ levels in astrocytes but not in microglia. MC4R stimulation triggers ERK-1/2 activation [9] and it is known that PPARs are targets of mitogen-activated protein kinases (MAPKs) (ERK-1/2 and p38 -MAPK) and PKA, among other kinases, and that the consequences of phosphorylation are varied and depend on many factors such as cellular context, receptor isotype, the kinase involved and the affected residue [37]. Furthermore, Guntur et al. have described a mechanism by which a kinase modulates PPAR-γ expression, without altering its mRNA levels [36]. All together, these data support the possibility that ERK-1/2 signaling may be mediating the stimulatory effect of NDP-α-MSH on PPAR-γ protein expression in astrocytes.

In microglial cells, we found that ERK-1/2 signaling may be involved, at least in part, in the inhibitory effect of NDP-α-MSH on PPAR-β expression. Both cAMP and PKA activators have been shown to increase PPAR-β activation [56] but so far there is no evidence that ERK-1/2 signaling modulates PPAR-β activity. There is however evidence of an inhibitory effect of PPAR-β activation on ERK-1/2 signaling [57], [58]. Further work will be necessary to better understand the cross-talk between PPARs and ERK-1/2 pathways in MC4R signaling in microglial cells.

As for the pathways involved in the effect of NDP-α-MSH on PPAR-γ expression in microglia and on PPAR-β expression in astrocytes, it is important to note that ERK-1/2 is only one of the possible downstream mediators of MC4R signaling. The activity of AMP-activated kinase, c-jun kinase, phosphatidylinositol-3-kinase and protein kinase C has also been shown to be modified upon MC4R activation [59] and there is evidence that PPAR-γ is a potential target of all of these kinases [60]. Plus, MC4R activation induces production of cAMP and stimulates the cAMP-PKA-CREB pathway [16] and, as mentioned before, cAMP and PKA activators have been shown to increase PPAR-β activation [56]. Thus, it is possible that one or more of these pathways may also be involved in the modulation of PPAR expression in our model.

α-MSH has been proposed as a useful treatment for CNS inflammatory diseases such as EAE, where it promotes the reduction of brain infiltrates, the inhibition of pro-inflammatory cytokines and the induction of IL-10 and TGF-β [61], [62]. Our present data indicate that NDP-α-MSH treatment increases IL-10 release from microglia, but not from astrocytes, and that this effect is not seen in the presence of the MC4R antagonist HS024, further supporting a role for this receptor in the observed effect of NDP-α-MSH. IL-10 is an anti-inflammatory cytokine that is upregulated by inflammatory stimuli in several cell types and is a key player in the resolutory phase of the inflammatory response [63]. It has the ability to suppress the release of NO, IL-6 and TNF-α induced by LPS in mixed astrocyte and microglial cultures [25] and α-MSH stimulates IL-10 release in monocytes and macrophages [26], [27]. Thompson et al. demonstrated the presence of a functional PPAR response element (PPRE) in the human IL-10 promoter region and the upregulation of this cytokine by a PPAR-γ agonist in dendritic cells [28]. Here, we show that the stimulating effect of NDP-α-MSH on IL-10 release in microglia is impaired in the presence of the PPAR-γ antagonist GW9662. However, we also found that the PPAR-γ antagonist reduced IL-10 release *per se* in microglia. Similarly, Zapata-Gonzalez et al. found that GW9662 decreased IL-10 release *per se* in dendritic cells [64]. Overall, our results indicate that PPAR-γ is involved in basal IL-10 production in microglial cells but given the inhibitory effect of the antagonist we were unable to asses whether the stimulatory effect of NDP-α-MSH is also exerted via PPAR-γ activation. Notwithstanding, it is possible that this receptor may be involved in NDP-α-MSH induction of IL-10 in microglial cells.

Finally, we show that NDP-α-MSH induces TGF-β release from astrocytes, but not from microglia. TGF-β is a cytokine that exerts multiple functions ranging from regulation of cell proliferation, development and migration, to modulation of inflammatory responses and CNS homeostasis [29]. This cytokine is able to inhibit the LPS-induced expression of TNF-α in astrocytes and microglia [30], [31]. Also, *in vitro* studies have shown that TGF-β can protect neurons from cell death induced by several neurotoxic factors and that the neuroprotective effects of TGF-β are mediated by glial cells [32]. Hence, NDP-α-MSH-induced TGF-β could be acting on neurons and on microglia, as it could also be acting in an autocrine fashion on astrocytes themselves. Accumulating evidence suggests that an interplay between PPAR-γ and TGF-β contributes to the regulation of cell proliferation, differentiation and their associated cellular functions [65]. In our model, the stimulatory effect of NDP-α-MSH on TGF-β release was not modified by the PPAR-γ antagonist GW9662, which suggests that PPAR-γ may not be involved in the effect of NDP-α-MSH on TGF-β release. However, PPAR-γ can also be activated in a ligand-independent fashion that may not be inhibited by GW9662, as described before [66]. Therefore, we cannot rule out the possibility that NDP-α-MSH may be inducing TGF-β release through PPAR-γ via a ligand-independent mechanism.

The differential effects of NDP-α-MSH on IL-10 and TGF-β release may be attributed to the cell-specific context in which MC4R activation takes place. Many different factors intervene in the receptor’s signaling, so that the cell-specific effects that we observe may be understood as the result of complex interplay between these factors. For instance, it has been suggested that MC4R may couple to G proteins other than Gs, such as Gi/o or Gq, thereby activating a completely different variety of signaling pathways, and that this alternative G protein coupling may be dependent on the ligand, the cell type involved and the level of MC4R expression [59]. The latter two factors may be of paramount relevance for understanding the differential responses obtained using the same stimulus in our two primary culture systems. We have reported that both α-MSH (the natural endogenous hormone) and NDP-α-MSH (its synthetic analogue) increase cAMP in a similar amount in astrocytes [16]. However, in view of the facts discussed above, we cannot discard the possibility that the endogenous agonist might lead to different intracellular events than those we observed using the synthetic ligand.

In summary, we show that production of two broad spectrum anti-inflammatory cytokines such as IL-10 and TGF-β and modulation of PPAR expression are triggered upon glial MC4R activation. We also propose that these mechanisms may be involved, at least partially, in the anti-inflammatory properties of melanocortins broadly reported in several models of neuroinflammation and neurodegeneration, contributing to ensure the expression of an effective anti-inflammatory program. Collectively, we provide new evidence supporting glial MC4R as a promising target for the development of therapeutics for the treatment of neuroinflammatory and/or neurodegenerative diseases.
